# 

**Published:** 1915-05

**Authors:** 


### ANNOUNCEMENTS.

DR. RALPH E. LUTHER.—Died February 12th at St. Luke's Hospital, Chicago, Ill., from pneumonia, in his thirty-ninth year, Ralph E. Luther, D.D.S. of Batavia, N. Y.

Dr. Luther was born in Warsaw, October 26, 1876. He was a graduate of the Warsaw High School, and Dental Department of the University of Buffalo, class of 1900, and in September of the same year commenced the practice of dentistry in Batavia.

He was married to Miss Marion Duncan in 1901, and is survived by his wife, two sons, Duncan and John, his mother, Mrs. Mary E. Luther, two brothers, Kendrick of Syracuse, and Guy S. of Schenectady, and a sister, Mrs. Roy McGregory of Batavia.

Dr. Luther was a charter member of Mu Chapter Xi Psi Phi fraternity, and had served as Deputy Supreme President. He was a member and past president of the Eighth District and Batavia Dental Societies, and a member of the Dental Society of the State of New York, and chairman of the committee on Oral Hygiene, the National Dental Association and Rochester Dental Society. He was also a member of the Masonic fraternity, and various other local organizations, but was particularly active in affairs of the Young Men's Christian Association, and Boy Scouts, where he rendered much valuable service by his untiring energy and enthusiasm.

Dr. Luther took a prominent part in matters connected with Oral Hygiene, and was one of the pioneers of the work in this state. He had made a specialty of pyorrhea work, and just prior to his death had completed a course in Chicago. Dr. Luther was a man of fine ability as an operator, and gave much promise of a brilliant future. He was an upright, conscientious, high-class professional man, who observed all the finer rules of conduct towards his patients and the profession. He was a splendid type of a high-toned professional gentleman. In his death the profession loses an intelligent, able and indefatigable worker, and his family and friends, a kind, generous and lovable companion and friend. His loss will be deeply mourned by a large circle of friends and acquaintances, who honored him for his many attractive qualities of heart and mind.

H. J. Burkhart.

---

DR. H. B. McFADDEN.—We are grieved to announce that Dr. H. B. McFadden, Treasurer of the National Dental Association died at his home in Philadelphia, February 14, 1915. Dr. McFadden has been prominent in dental society work for many years and his untimely death will be a great regret to his many friends.

---

Dr. W. Xavier Sudduth, at one time a prominently known dentist, teacher, and scientist died at his home in Roundup, Montana. Dr. Sudduth was Professor in the Dental School of the University of Minnesota and Dean of this School from 1890 to 1895. He did a considerable amount of excellent research work. He wrote the chapter on Embryology and Dental Histology for the American System of Dentistry. He made a considerable study of hypnotism and used it extensively in his practice for relieving dental pain. He quit practice and teaching and went west and became extensively interested in growing alfalfa and other grasses for cattle foods. He put his scientific training to good use in studying this problem, and became very successful.

---

THE TENNESSEE STATE ASSOCIATION will meet in Sewanee, June 24th to 26th.

THE MICHIGAN STATE BOARD for registration to practice in that state will meet in Ann Arbor, June 14th. Write Dr. A. W. Haidle, Negaunee, Mich., for particulars.

---

THE KENTUCKY STATE DENTAL ASSOCIATION will meet in Ashland, June 8th-10th.

---

ANNUAL MEETING OF THE MEMBERS OF THE DENTAL PROTECTIVE ASSOCIATION OF THE UNITED STATES. The regular annual meeting of the members of the Dental Protective Association of the United States was held at the LaSalle Hotel in the city of Chicago on Monday afternoon, Dec. 21, 1914. Due notice in writing was given to each member of the Association of this meeting in compliance with the requirements of the by-laws, and notification was also published in the daily press. The president Dr. J. G. Reid, presided. An unusually large number of the members was present but in order to give those who were unable to attend some idea of the present status of the Association those at the meeting proposed that a brief synopsis of the secretary's and treasurer's report be published in one or more publications of general circulation among dentists.

The secretary, Dr. J. P. Buckley, presented a detailed report of the activities of the officers and directors during the year, described the organization of the board, the election of the officers and the taking over of the books and records of the Association from the administrative officers of the preceding years. He commented particularly upon the thorough, accurate and business-like manner in which the former president, Dr. J. N. Crouse, had kept the books and records of the Association and carried on the voluminous correspondence entailed by the agreement entered into with Dr. W. H. Taggart on behalf of the Association.

The Association has now about eight thousand members upon its books, nearly five thousand of whom paid the special assessment in 1900 while over forty-two hundred availed themselves during 1910 to 1912 of the agreement entered into with Dr. Taggart and paid the requisite fifteen dollars.

The officers of the Association increased the annual revenue by changing the investments during the year in purchasing twenty-one thousand dollars ($21,000) of municipal bonds of the highest grade at exceptionally low prices, as the bonds were purchased at the time when the market was at the lowest ebb, due to the foreign war. About five thousand dollars in cash remains on deposit at a low rate of interest in substantial savings banks in the city in charge of Dr. D. M. Gallie, treasurer, who has given a Surety Company's bond to the Association in the sum of five thousand dollars. The directors during the year amended the by-laws so as to require that the securities and other valuable papers of the Association be deposited in a box rented in the name of the Association in one of the large safety deposit company's vaults with the requirement that the box may be opened only in the presence of two members of the Board.

Certain doubtful assets which have remained stagnant for many years were converted and are being liquidated in a satisfactory manner so that the finances of the Association are in excellent condition.

The secretary further called attention to the statement which he caused to be published in the Bulletin of the National Dental Association in the October issue emphasizing the fact that the Dental Protective Association of the United States was still in active business, had more than twenty-five thousand dollars ($25,000) in the treasury and was prepared at all times to carry out the purpose of its organization. To this published statement of October last the secretary now directs the attention of all members who are still in doubt as to their status under the Taggart agreement.

From the treasurer's report for the year ending December 10, 1914, the following facts may be mentioned:

The treasurer received as a cash balance on hand at the beginning of the year twenty-five thousand, six hundred twenty-eight dollars and thirty-five cents ($25,628.35). The receipts during the year from all sources amounted to two thousand thirty-six dollars and seventy-eight cents ($2,036.78). The disbursements during the year were two hundred forty-seven dollars and five cents ($247.05) exclusive of the purchase of twenty-one thousand dollars ($21,000) of bonds, which amounted to a change merely in the form of the assets. The total assets of the Association on December 19, 1914, amounted to thirty-five thousand two hundred eighty-seven dollars and fifty-eight cents ($35,287.58), of which twenty-seven thousand two hundred eighty-seven dollars and fifty-eight cents ($27,287.58) was in municipal bonds and cash and eight thousand dollars ($8,000) in individual notes secured by a corporation note for three times the amount.

The present Board of Directors were re-elected for the ensuing year.

(*Signed*)

J. G. Reid,
J. P. Buckley,
D. M. Gallie,
Board of Directors.

---

STATE BOARDS OF EXAMINERS' MEETINGS.

California Board of Examiners, San Francisco, June 4th; Los Angeles, June 18th. District of Columbia Board of Examiners, Washington, June 7th to 10th. Illinois Board of Examiners, Chicago, June 10th. Indiana Board of Examiners, Indianapolis, June 14th to 19th. Iowa Board of Examiners, Iowa City, June 7th. Maine Board of Examiners, Augusta, July 1st to 3d. Maryland Board of Examiners, Baltimore. May 27th and 28th. Michigan Board of Examiners, Ann Arbor, June 14th to 19th. Montana Board of Examiners, Helena, Mont., July 12th to 15th. Nebraska Board of Examiners, Lincoln, June 9th. New Hampshire Board of Registration, Manchester, June 14th 12th. South Carolina Board of Examiners, Columbia, June 28th to 30th. North Carolina Board of Examiners. Wrightsville Beach, June 21st. Oklahoma Board of Examiners, Oklahoma City, June 14th. Pennsylvania Board of Examiners, Philadelphia and Pittsburgh, June 9th to 12th. South Carolina Board of Examiners, Columbia, June 15th. Texas Board of Examiners, Dallas, June 21st. Vermont Board of Examiners, Montpelier, June 28th to 30th. Virginia Board of Examiners, Richmond, June 8th. West Virginia Board of Examiners, Charleston, June 9th. Wisconsin Board of Examiners, Milwaukee, June 21st.

---

### MEMORIAL RESOLUTIONS.

Professor James Truman, one of the most distinguished of the Associate members of the American Academy of Dental Science, died at his home in Philadelphia, November 26, 1914 in the eighty-eighth year of his life. The loss of Professor Truman will be deeply mourned wherever dentistry is practiced. He was one of the founders of his profession, and one of the best exponents of its larger possibilities. He was also one of our foremost teachers and the students who sat under his instruction reverenced him as they would a father. All who came in contact with Dr. Truman, realized the exalted nature of the man and his nobility of character, his love of mankind and his charity for all. His was a fully rounded life, beginning early with large promise, equalling every anticipation in its maturity, fertile and beautiful to its close in the ripeness of its well-filled years.

Dr. Truman began the study of dentistry with his father who was both a dentist and a physician. He graduated at the Philadelphia College of Dental Surgery in 1854. In 1864 he accepted the position offered him as demonstrator-in-chief, of operative dentistry in the college from which he graduated. In 1865 Dr. Truman was elected to fill the chair of Dental Physiology and Operative Dentistry in the Pennsylvania Dental College, and he held that position until 1870, when he resigned. He was the editor of the *Dental Times*, during this professorship, and during the four years of its existence, the productions of his pen were published in this journal. On account of his health, he went to Germany and settled at first in Frankfort practicing a year there. He then went to Hanover, and had among his patients many of the nobility and the wealthy residents of that province. In 1880 he returned to America and began practice in Philadelphia again. In 1882 he was elected Professor of Dental Pathology, Therapeutics, and Materia Medica in the Department of Dentistry of the University of Pennsylvania. In 1883 he was made Secretary, and subsequently Dean, which position he held until he retired in 1896. In 1890 he was the editor of the *International Dental Journal,* and he held that position until the publication ceased in 1905. He received the degree L.L.D. from the University of Pennsylvania in 1904. Professor Truman was one of the pioneers of organized professional dentistry and brought to the solution of this problem a commanding personality, a vigorous, and at times an aggressive intellectuality, a masterful command of language, and a dignity and forcefulness of mind which inevitably carried conviction to his hearers. We feel that no tribute to his memory can be too generous or too universal, therefore

*Be it Resolved,* That in the death of Professor Truman, the American Academy of Dental Science loses one of its most distinguished fellows, who has been a signal honor to his profession, whose life was full of simplicity, tenderness and personal charm, whose advanced years were as beautiful as his manhood and his youth, a man who was loved wherever known.

R. R. Andrews,

Edward C. Briggs,

T. O. Loveland,

*Committee.*

---

Dr. Louis Jack, a distinguished associate fellow of the American Academy of Dental Science, died at his home at Moyland, Pa., near Philadelphia, on December 9, 1914, in his eighty-third year. He was born at Germantown, a suburb of Philadelphia, March 26, 1832, and enjoyed a practice of fifty-four unbroken years in his chosen profession.

At an early age he was taken by his parents to Beaver County, Pa., where he received his preliminary education at the Bridgewater Academy. At the age of twenty he returned to Philadelphia to look around to see what he might find to do and soon decided to take up the study of dentistry. He first became associated with Dr. William R. White in whose laboratory he was employed, and afterwards with Dr. C. C. Williams. It was at this time that he learned that the Philadelphia College of Dental Surgery, the first school in Pennsylvania to teach dentistry, was about to open its doors to students and he was the first matriculate to register. (September 2, 1852). He graduated in a class of nineteen on the twenty-eighth day of February, 1854.

Soon after graduating, he opened his first office in the house of Dr. Robert Arthur and was closely associated with him. It was during the winter of 1855 that the cohesive property of gold foil was first brought to light in Dr. Arthur's laboratory.

He was instructor at the College for several years after his graduation and in 1857 moved his office to Germantown within a stone's throw of the spot where he was born and in 1864 he returned to the city proper where he remained until his retirement in 1908.

For several years during the early seventies, he devoted much of his time after office hours to the construction of an electric mallet. This invention he gave to the dental profession and for this act the Odontographic Society of Pennsylvania presented to him a testimonial of thanks for his "Professional Liberality and Loyalty to Professional Ethics."

He early recognized the importance of a good and lasting school for the teaching of dentistry in this country, was of the first to urge the institution of such a department at the University of Pennsylvania and was instrumental in the formation of that department in 1877 and in which he was an occasional lecturer.

He was the "Father member" of the Philadelphia Dental Club having been a member covering a period of forty-two years, from its origin in 1872 until the time of his death.

He was interested in the development of the *International Dental Journal,* and was for a time president of the corporation which owned and published it. He was a member of the National Dental Association, the American Academy of Dental Science, the Odontographic Society of Pennsylvania, the Odontological Society of Pennsylvania, the Pennsylvania State Dental Society, the Academy of Stomatology, and the Philadelphia Dental Club.

Dr. Jack was the last survivor of the class of 1854 of the old Philadelphia College of Dental Surgery. He was a classmate of Prof. James Truman, who died in Philadelphia, only a month before.

Dr. Jack was a man of unusual ability, a gentleman of the highest character, dignified and refined. He was a sturdy advocate of all that was best in his profession. The hand of an artist showed itself unmistakably in every thing he did. His skill as an operator gave to him an international reputation. The Academy honors the memory of Dr. Louis Jack, and sorrowfully adds one more illustrious name to its memorial records.

He did much to make our profession what it is today, one of the great ameliorating agencies of modern civilization.

He was not untimely taken. His life was prolonged many years, happy and famous.

We look upon his distinguished attainments with feeling of gratitude and appreciation. No man ever exercised a more genial personal influence over his friends, and those who knew him best realized the exalted character of the man, and loved him. Therefore

*Be it Resolved,* That in the death of Dr. Louis Jack the Academy mourns one of its distinguished fellows, who has ever been an honor to his profession and we deem it fitting to make a record of our sense of sorrow, at his loss.

ROBERT R. ANDREWS,
CHARLES A. BRACKETT,
EUGENE H. SMITH,
*Committee.*

